# Caring for Hospitalized COVID-19 Patients: From Hypes and Hopes to Doing the Simple Things First

**DOI:** 10.4269/ajtmh.21-0961

**Published:** 2021-11-01

**Authors:** Marcus J. Schultz, Ni Ni Tun, Gentle S. Shrestha

**Affiliations:** ^1^ Mahidol–Oxford Tropical Medicine Research Unit (MORU), Mahidol University, Bangkok, Thailand;; ^2^ Amsterdam University Medical Centers, AMC, Amsterdam, The Netherlands;; ^3^ Nuffield Department of Medicine, University of Oxford, Oxford, United Kingdom;; ^4^ Myanmar–Oxford Clinical Research Unit (MOCRU), Yangon, Myanmar;; ^5^ Medical Action Myanmar (MAM), Yangon, Myanmar;; ^6^ Department of Anaesthesiology, Tribhuvan University Teaching Hospital, Kathmandu, Nepal

COVID-19 has been haunting the world for more than 18 months, and there seems to be no end to the disaster that is continuing to unfold. The pandemic is killing countless people every day worldwide; therefore, we have to address the ongoing and unprecedented challenges for all health systems. There is no prospect of a near-term end because the world seems to forget that there is an absolute need for high-coverage vaccination that needs to be universal, including in resource-limited settings.

We have learned much about this “new” disease during the ongoing pandemic. During the earliest stages of the outbreak, the unknown nature of the new virus meant that it presented a terrible threat to all health systems. However, over time, health systems have adapted, and we have witnessed a significant increase in both our understanding of the disease and the introduction of nonspecific but appropriate treatments, including dexamethasone and other inhibitors of inflammation for hospitalized COVID-19 patients.

In this issue of *American Journal of Tropical Medicine and Hygiene*, Abd-Elsalam and others report a randomized clinical trial of remdesivir.^1^ During this study performed in Egypt, patients were randomly assigned in a 1:1 ratio to receive either standard care plus remdesivir for 10 days or standard care alone. Although remdesivir did not prevent death, it reduced the hospital length of stay (median of 10 days versus 16 days; *P* < 0.001). Obviously, this latter finding is important because of the extreme shortages of hospital beds that can arise during local outbreaks of COVID-19, and also because this strategy has the potential to be a cost-effective intervention.^2^ However, it is uncertain why remdesivir was administered for 10 days instead of 5 days, as in other studies.^3^ Might a shorter course have resulted in comparable findings?

Of note, the investigators did not use predefined criteria for hospital discharge, and their open-label design could have induced bias. It is interesting to note what standard treatment consisted of, including unproven interventions such as zinc, acetyl cysteine, lactoferrin, and vitamin C. Using these agents while considering the applicability of the study to a resource-limited setting seems a bit contradictory. The use of steroids was not mentioned, and prophylactic use of anticoagulation was only mentioned as “when indicated.” Therefore, the real-world situation in Egypt included management of COVID-19 with unproven interventions and, it appears, inadequate use of proven interventions.

Should remdesivir be considered for COVID-19 in settings where resources, including hospital beds, are scarce? Currently, the evidence is against remdesivir. For instance, in the large RECOVERY trial,^4^ death occurred for 10.9% of patients receiving remdesivir and 11.1% of control patients (*P* = 0.50). More importantly, the RECOVERY trial also showed no benefit with regard to the hospital length of stay, although the investigators noted that “patients who would be considered fit for discharge might be kept in the hospital somewhat longer just because they were being given a trial drug.” Could it be that patients with different features respond differently to this treatment, and, if so, are COVID-19 patients in Egypt different from elsewhere? Answers to these questions are urgently needed.

Healthcare providers should not forget the proven benefits of simple specific pharmacological interventions, notably dexamethasone for noncritically ill as well as critically ill COVID-19 patients,^5^ and anticoagulation in patients not in need of critical care.^6^^,^^7^ Both dexamethasone and anticoagulation are relatively inexpensive and of great interest in resource-limited settings. However, we should realize that even these “cheap” treatments come with challenges. Despite the growing evidence of greater effectiveness of low-molecular-weight heparin compared with unfractionated heparin for the prevention of venous thromboembolism in critically ill patients,^8^ because of the higher cost of the former, use of unfractionated heparin often remains a more practical option. For instance, in late July 2021, during a surge of COVID-19 cases, Myanmar faced the huge problem of drug shortages, including dexamethasone and unfractionated heparin, because of high prices, pharmacy closures, and restrictions on the importation of drugs.

**Figure 1.**
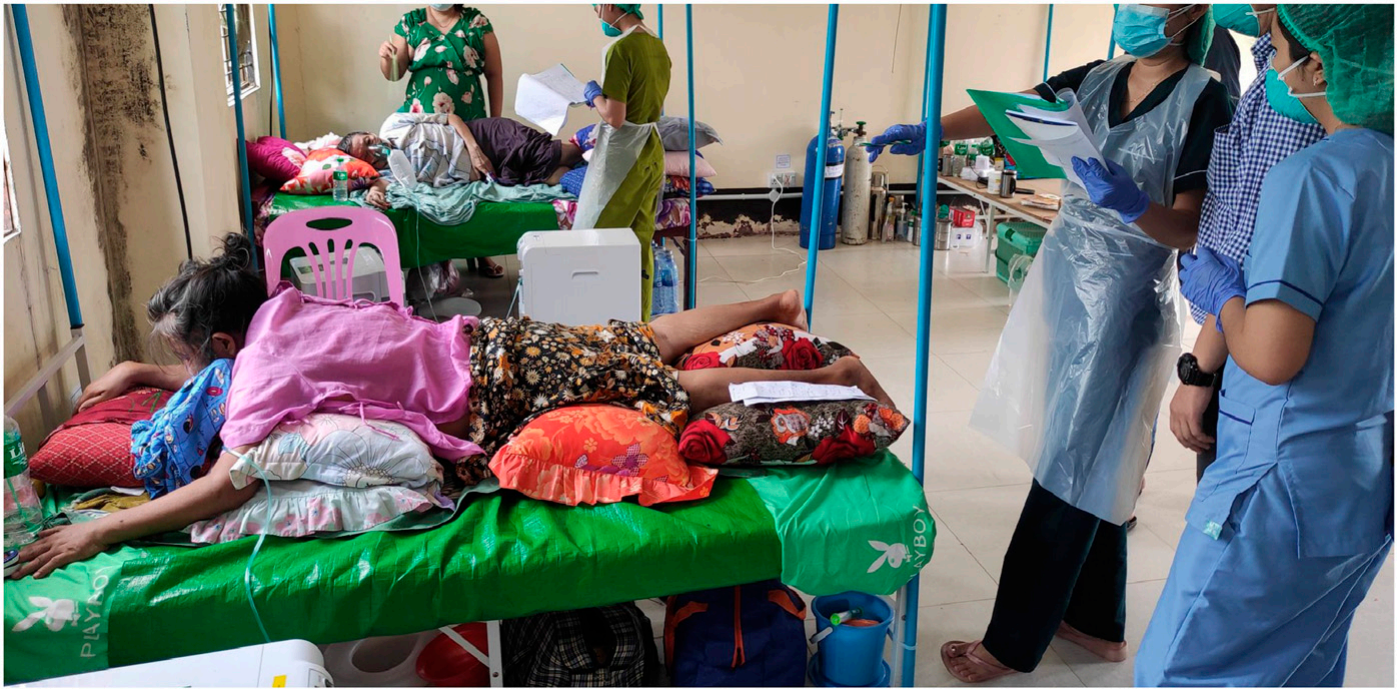
An awake COVID-19 patient placed in the prone position in a charity hospital in Myanmar. Photo courtesy of Dr. Ni Ni Tun. This figure appears in color at www.ajtmh.org.

One important, if not the most common, reason for the hospitalization of COVID-19 patients is the need for supplementary oxygen. Naturally, healthcare providers should also focus on this challenging aspect of care because the world is faced with extreme and unprecedented oxygen shortages.^9^ Oxygen must be supplied “sufficiently” but, above all, “economically,” and oxygen-sparing strategies such as awake prone positioning should be considered for every COVID-19 patient (Figure 1).^10^^,^^11^ The use of checklists to rationalize the use of oxygen and to minimize wastage can be very helpful when oxygen resources are limited.^12^ In Myanmar, oxygen cylinders and concentrators are not available for many severe COVID-19 patients. Limited refilling of cylinders and frequent power outages while using concentrators made proper care of these patients almost impossible. Finally, for patients who eventually need invasive ventilation, lung-protective ventilation, which consists of using low tidal volumes^13^^,^^14^ and avoiding high airway pressures,^15^ should be practiced. For those patients, prone positioning can also be effective, and sessions should be sufficiently long to reduce the burden for the intensive care unit staff.^16^

One important notion is that dexamethasone and anticoagulation had dissimilar effects on the outcomes of patients on general wards compared to patients in the intensive care unit (ICU). This is, of course, a challenge in settings where the distinction between a non-ICU patient and an ICU patient is unclear, as in the aforementioned studies. This distinction can be further blurred during case surges when ICU beds are fully occupied, thus mandating the management of sicker patients in the emergency department or on general wards, including those receiving respiratory support measures such as noninvasive ventilation. Additionally, the quality of care and infrastructure of makeshift ICUs may remain suboptimal.^17^

The findings of Abd-Elsalam and others are of high interest and should trigger new trials; evidence is urgently needed because the available data are too limited to change existing guidelines. Potential benefits of potential interventions must be balanced against additional costs and toxicities.^18^ Remdesivir is not exempt from this consideration. Finally, let us not forget “to do simple things first,” where “simple” means “affordable,” “available,” “safe,” and “feasible” care. This approach should be the standard for COVID-19 and other serious illnesses, always and everywhere, in resource-rich and resource-limited settings alike.
